# The complexity of eye-hand coordination: a perspective on cortico-cerebellar cooperation

**DOI:** 10.1186/s40673-020-00123-z

**Published:** 2020-11-13

**Authors:** John-Ross Rizzo, Mahya Beheshti, Tahereh Naeimi, Farnia Feiz, Girish Fatterpekar, Laura J. Balcer, Steven L. Galetta, Aasef G. Shaikh, Janet C. Rucker, Todd E. Hudson

**Affiliations:** 1 Department of Rehabilitation Medicine, NYU Grossman School of Medicine, New York, NY USA; 2 Department of Neurology, NYU Grossman School of Medicine, New York, NY USA; 3 Department of Biomedical Engineering, NYU Tandon School of Engineering, New York, NY USA; 4 Department of Mechanical & Aerospace Engineering, NYU Tandon School of Engineering, New York, NY USA; 5 Department of Radiology, NYU Grossman School of Medicine, New York, NY USA; 6 Department. of Ophthalmology, NYU Grossman School of Medicine, New York, NY USA; 7 Department of Population Health, NYU Grossman School of Medicine, New York, NY USA; 8 Department of Neurology, University Hospitals Cleveland Medical Center and Louis Stokes Cleveland VA Medical Center, Case Western Reserve University, Cleveland, OH USA

**Keywords:** Eye-hand coordination, Visually-guided reaching, Cerebellar stroke, Cortical stroke

## Abstract

**Background:**

Eye–hand coordination (EHC) is a sophisticated act that requires interconnected processes governing synchronization of ocular and manual motor systems. Precise, timely and skillful movements such as reaching for and grasping small objects depend on the acquisition of high-quality visual information about the environment and simultaneous eye and hand control. Multiple areas in the brainstem and cerebellum, as well as some frontal and parietal structures, have critical roles in the control of eye movements and their coordination with the head. Although both cortex and cerebellum contribute critical elements to normal eye-hand function, differences in these contributions suggest that there may be separable deficits following injury.

**Method:**

As a preliminary assessment for this perspective, we compared eye and hand-movement control in a patient with cortical stroke relative to a patient with cerebellar stroke.

**Result:**

We found the onset of eye and hand movements to be temporally decoupled, with significant decoupling variance in the patient with cerebellar stroke. In contrast, the patient with cortical stroke displayed increased hand spatial errors and less significant temporal decoupling variance. Increased decoupling variance in the patient with cerebellar stroke was primarily due to unstable timing of rapid eye movements, saccades.

**Conclusion:**

These findings highlight a perspective in which facets of eye-hand dyscoordination are dependent on lesion location and may or may not cooperate to varying degrees. Broadly speaking, the results corroborate the general notion that the cerebellum is instrumental to the process of temporal prediction for eye and hand movements, while the cortex is instrumental to the process of spatial prediction, both of which are critical aspects of functional movement control.

## Introduction

Eye–hand coordination (EHC) is a complicated process that requires the precise activation of ocular and manual motor systems. Timely and skillful movements such as reaching for and grasping small objects depend on the acquisition of high-quality visual information about the environment and simultaneous eye and hand control [1]. When reaching for or touching objects in our three-dimensional world, the eyes will typically fixate objects of interest before any hand movement begins [2]. Rapid eye movements called saccades are responsible for this fixation by shifting gaze from one part of the visual scene to another, in turn bringing the object of interest into focus on the fovea, the portion of the retina with highest visual acuity. Normal reaching is characterized by a movement sequence in which saccades are tightly synchronized to arm movement. This temporal link between eye and hand movement supports coordination and functional performance by bringing gaze to the target of the arm movement at precisely the moment that this high-definition visual information is most useful.

Multiple areas in the brainstem, basal ganglia and cerebellum, as well as some frontal and parietal structures, have critical roles in the control of saccades and EHC [3]. In particular, extensive evidence supports the cerebellum playing a key role in the coordination of eye and hand during pointing, tracking, and reaching tasks. This is not a new idea; its foundation dates back to 1824 when Flourens observed that after cerebellectomy in the pigeon, “the will, the senses, the perception remained, but the coordination of movement, the ability for controlled and determined movement, was lost” (Flourens 1824) [4]. The cerebellum is known to support motor control, coordination, motor learning, temporal prediction and timing [1, 5, 6]. It is often said that the cerebellum provides a “forward model” of the hand, incorporating input on the current state of the motor system along with the intended goal in order to assist with planning of the next motor command. These models underscore the cerebellum’s role in EHC, as predictions about the consequences of descending motor signals are required to orchestrate cooperation between motor effectors [1, 5] and to optimize the timing and precise spatial goals of saccadic eye movements, thereby facilitating fluid motor control in dynamic environments [3, 7, 8]. Lesions of the cerebellum can yield increased saccade latencies [9, 10], as well as saccadic intrusions [11, 12]. In addition, patients with cerebellar lesions affecting the fastigial oculomotor region tend to show ocular motor dysmetria (e.g., hypermetria and hypometria) [3]; this may be related to mis-timed saccade termination. These saccadic disturbances have known and characterized downstream effects on hand control and perturb the cooperation between effectors [13].

On the other hand, the role of the cerebral cortex in eye-hand movements is as complex as EHC itself, as many regions and subregions are involved in this process. The most significant cortical functions involved in EHC are visuospatial processing, re-mapping spatial information after eye movements, and transferring motor commands to primary motor areas for implementation [14]. The cortex processes visuospatial information and controls the complex signal generation required for multi-joint muscular contractions during accurate reaching and grasping [14], as well as houses the cortical network of visuomotor areas for controlling saccades [15]. Commensurate with the prominent cortical role in spatial processing, cortical lesions affecting EHC tend to cause spatial reach errors and impair eye-hand accuracy [16]. Although both cortex and cerebellum contribute critical elements to normal eye-hand function, our hypothesis is that differences in these contributions suggest that there may be separable deficits following injury [17]. In order to preliminarily test this hypothesis for this perspective we compared eye and hand-movement control in a patient with cortical stroke relative to a patient with cerebellar stroke, with a particular emphasis on the dissociation of eye and hand movements during reaching. We hypothesized that cortical stroke would cause greater spatial coupling deficits and that cerebellar stroke would more greatly impair temporal coupling during a visually-guided reaching task.

## Methods

### Participants

Two patients with a history of stroke were selected for study due to their self-report of eye-hand dyscoordination following stroke. We specifically selected one such patient with a history of cerebellar stroke (Case 1), and a second patient with a history of cortical stroke (Case 2) in order to preliminarily investigate our hypothesis that eye-hand coordination deficits would differ based on general cerebellar versus cortical dysfunction. Both patients provided informed consent to study procedures that have been approved by the NYU Grossman School of Medicine Institutional Review Board.

### Assessments

At clinical presentation, a focused stroke history and neurologic-musculoskeletal examination were performed. The Fugl-Meyer Assessment [(FMA), a stroke-specific, performance-based assessment index] [18, 19] was completed. Both patients were able to perform reaching and pointing movements.

Visual impairments were assessed using the Beery Buktenica Developmental Test of Visual-Motor Integration [20, 21], by standard clinical tests for visual acuity (Snellen chart) and visual fields with confrontation testing. The 25-item National Eye Institute Visual Functioning Questionnaire (NEI-VFQ-25) was administered to quantify the disability extent due to perceived visual deficits and the Barthel Activities of Daily Living Index was also completed. In addition, the Nine Hole Peg (9HPT) and Box and Blocks tests (BNB) were administered to assess vision, eye-hand coordination, and manual dexterity [22–25]. The patient with cerebellar stroke also was assessed with the International Cooperative Ataxia Rating Scale (ICARS) [26] and Scale for Assessment and Rating Ataxia (SARA), which is a clinical scale to quantify the level of impairment resulting from ataxia [27].

### Experiment

**Fig. 1:**
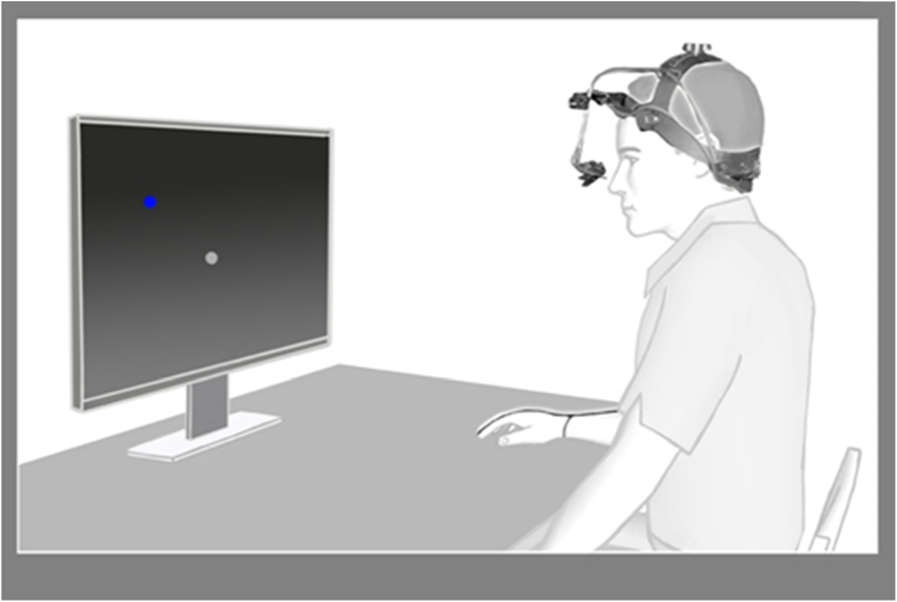
Schematic of experiment setup (monitor, tabletop, limb tracker and eye tracker)

Participants were seated facing 60 cm from a Dell 27″ monitor on which experimental tasks were displayed. A video eye tracker (EyeLink ll) was used to record eye movements. Participants were seated on an adjustable chair in front of a desk positioned immediately in front of the screen. The eye tracker was calibrated for each participant before each session. A magnetic-field based motion sensor (Polhemus) was affixed to the distal aspect of the index finger of the hand on the to-be-tested arm (Fig. 1).

A low-power magnetic field generator was affixed to the underside of the wooden desk holding the Dell monitor, and the Polhemus sensor was affixed to the finger by first placing it on the fingertip and securing it at three locations (proximal and distal phalanx and wrist). A nine-point grid on the desk spanning 12 by 9 cm was used to calibrate the Polhemus sensor within the magnetic field. At the beginning of each trial participants were asked to place their fingertip at known locations on the table to calibrate the sensor within the magnetic field along the tabletop.

Movements of the fingertip relative to the center of the table were detected by the Polhemus sensor and displayed onscreen (blue circle), as shown in Fig. 1. We started each recording session with 20 practice trials to familiarize participants with the demands of making reaching movements on the tabletop (participants were instructed to make arm movements as quickly as possible, as if attempting to ‘squash a bug’). Following this practice set, calibration and validation of the eye tracker were performed, at which point participants completed 25 experimental reaches. At the beginning of each reach, participants were required to simultaneously touch and fixate the start position (blue circle) at the center of the screen. Once this start configuration of the eye and hand was achieved, the “target” (a half-degree random ‘sparkle’) appeared. A beep cued the participant to initiate a combined look-and-reach movement to the target. Upon completion of each reach, feedback of the fingertip’s touchpoint appeared on the screen, giving explicit error feedback regarding reach accuracy.

### Statistical analysis

**Fig. 2:**
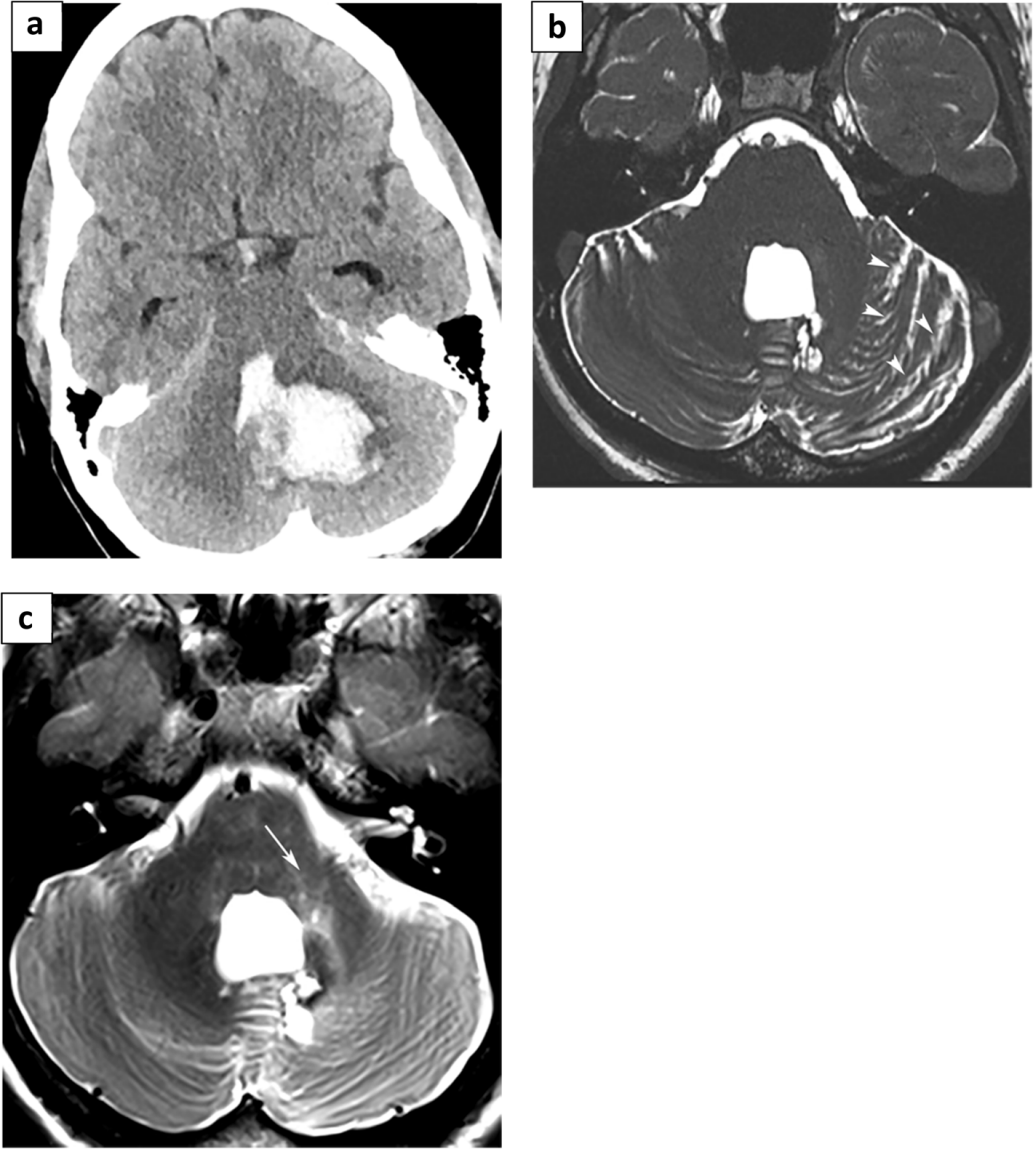
Neuroimaging in Cerebellar Stroke. **a** Non-contrast CT scan demonstrates intraparenchymal hemorrhage in the region of the quadrangular and simple lobules with secondary intraventricular extension. **b** Post-decompression of the hemorrhage, follow-up MRI demonstrates asymmetric prominence of the left cerebellar fissures (arrowheads) suggestive of underlying volume loss. **c** Axial T2WI demonstrates asymmetric bright signal in the region of the left brachium pontis with associated volume loss (arrow)

Data were median-filtered to remove outliers, and kinematic parameters were estimated after individual trials were aligned to the time of reach onset (spatiotemporal reach traces are plotted in Fig. 2). Velocity traces were unremarkable and were not studied further. Two-sample *t*-tests were used to compare pairs of means. The results were compared with Welch’s *t*-test due to unequal sample sizes and likely heteroscedasticity. As an adjunct to traditional *t*-tests, Bayesian analogues of the reported t-tests confirm our statistical results.

## Case descriptions

### Case 1: cerebellar stroke

A 43-year-old woman presented in 2015 with severe headache, followed by a sudden decreased level of consciousness. Neuroimaging revealed a cerebellar hemorrhage with mass effect, intraventricular extension, and hydrocephalus (Fig. 2).

She underwent suboccipital craniectomy and evacuation of intraventricular and cerebellar hemorrhages. She was discharged with residual ataxia, imbalance, dizziness, and intermittent binocular vertical diplopia. She was stabilized and discharged to an acute inpatient rehabilitation unit. The patient completed an acute inpatient course over approximately 3 weeks. Following her discharge home, physical, occupational, vestibular and speech therapy were initiated and continued over the next 2 years following her stroke.

Initial neuro-ophthalmic examination 3 months after her hemorrhage revealed normal visual acuity, pupillary reactivity, visual fields, and full eye movements. Frequent square wave jerks were present during fixation in central position. There was gaze-evoked nystagmus (right-beating in right gaze, left-beating in left gaze, and up-beating in up gaze) and rebound nystagmus upon return to central position. Horizontal and vertical saccades were hypermetric, horizontal and vertical smooth pursuit were saccadic, and VOR suppression was abnormal. Head impulse test was normal. By cross-cover and Maddox rod testing, there were small ocular misalignments (esophoria in left gaze; left hyperphoria in central and down gaze, right hyperphoria in up gaze and bilateral head tilts) that localized to left sixth and either bilateral fourths or an incomitant skew deviation. During a recent exam in 2019, findings were similar, with the exception of resolved square wave jerks and rebound nystagmus.

In her visit to our laboratory in 2018, approximately 3 years after her stroke, the patient reported difficulties with eye-hand coordination and mild visual problems that were not bothersome. The patient denied use of any medication with neurological mechanisms of action or side effects. Finger-to-nose testing was normal on the right side and abnormal on the left, as she was unable to follow a straight line with the left hand when reaching for a target. No dysdiadochokinesia was present. Her gait was slightly unstable and ataxic but with a narrow base. She was noted to lean slightly to the right with walking.

### Case 2: cortical stroke

**Fig. 3:**
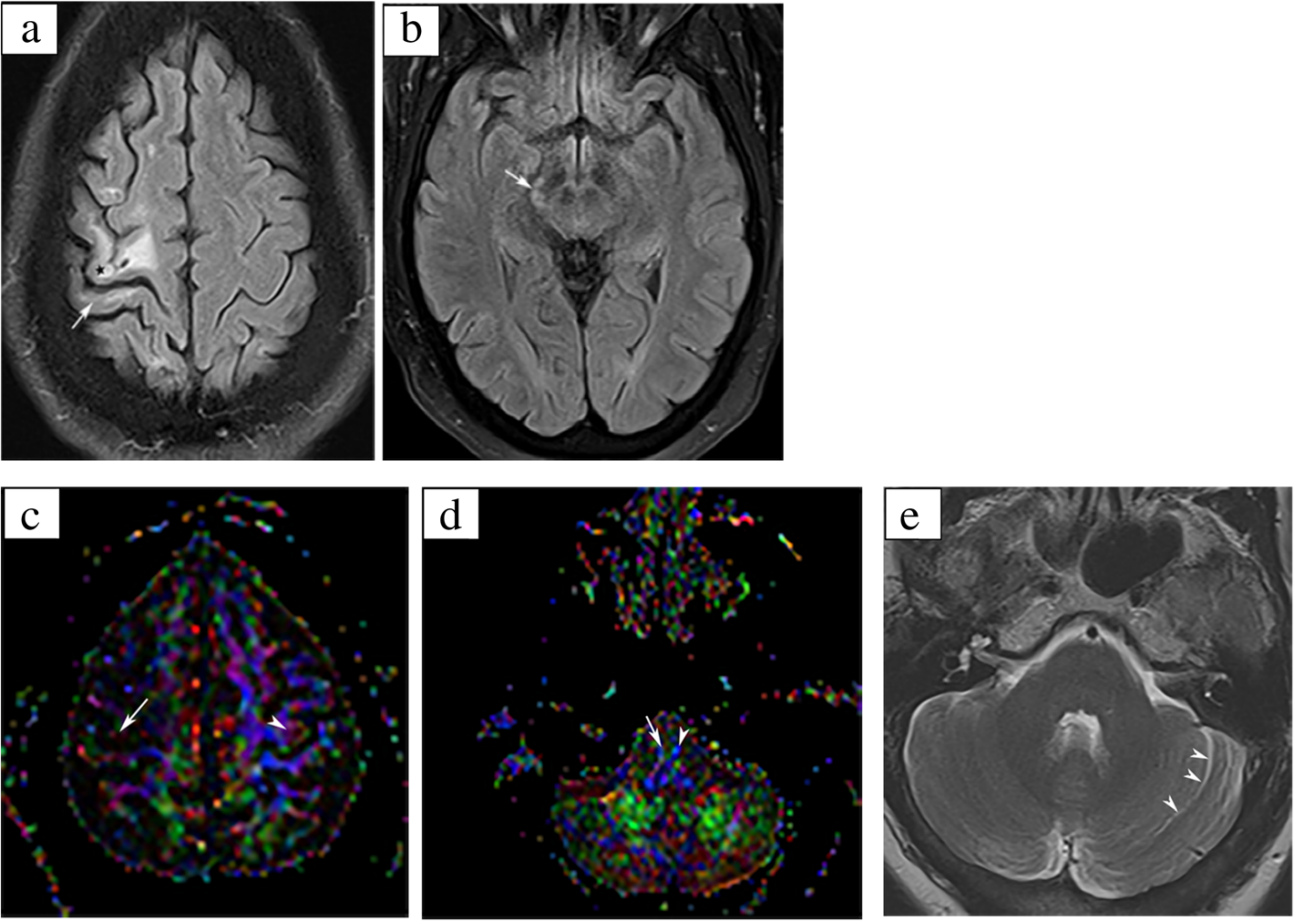
Neuroimaging in Cortical Stroke. **a** Axial FLAIR image demonstrates volume loss involving the right frontal lobe predominantly in the region of the precentral gyrus (star). There is also abnormal signal suggestive of volume loss along the anterior bank of the post-central gyrus (arrow). In addition, there are scattered areas of volume loss in the region of the middle frontal gyrus as well as the white matter of the superior frontal gyrus. **b** Axial FLAIR at the level of the midbrain demonstrates asymmetric bright signal along the right cortico-spinal tract (arrow) suggestive of ipsilateral Wallerian degeneration. **c** DTI FA map demonstrates loss of normal signal seen in the region of the right precentral gyrus (arrow) when compared to the left (arrowhead) . **d** DTI FA map at the level of the medulla demonstrates loss of normal bright blue color signal in the region of the right pyramid (arrow) when compared to the left (arrowhead) suggestive of Wallerian degeneration. **e** Axial T2WI demonstrates asymmetric prominence of the left cerebellar fissures (arrowheads) suggestive of underlying volume loss possibly related to crossed cerebellar

A 52-year-old woman with a history of hypertension and diabetes mellitus presented in 2015 with severe progressive weakness in the left upper extremity. MRI revealed ischemic infarction of the right high frontal lobe (Fig. 3). She was treated, stabilized and discharged to an acute inpatient rehabilitation unit. Her primary impairment was left-sided weakness. The patient completed an acute inpatient course over approximately 3 weeks. Subsequently, outpatient physical, occupational, and speech therapy were initiated and continued for approximately 2 years following her stroke.

Initial clinical examination revealed normal visual acuities, pupillary reactivity, visual fields, and full eye movement range with no abnormal spontaneous eye movements. Cerebellar function was intact. There was a weakness of left intrinsic hand muscles and decreased left-arm swing during ambulation.

In her visit to our laboratory in 2018, approximately 3 years after her stroke, the patient reported difficulties with eye-hand coordination, as well as left-sided weakness involving the upper and lower extremities and impaired activities of daily living, inclusive of mobility as a result of ambulation difficulties. She reported taking oral hypoglycemic and antihypertensive medications for the past 3 years. Neuro-ophthalmic examination demonstrated normal visual acuities (with correction) at distance, symmetric and reactive pupils, and full eye movement range with no nystagmus or saccadic intrusions.

## Results

Table 1 summarizes assessment results for the two patients. The patient with cortical stroke needed a longer time to complete the 9HPT and performed much worse on the BNB task with her affected left side. In both tests, we observed that the prolongation in time was due to impaired grasping and manipulating the pegs and blocks, with relative sparing of the transfer phase. We did not observe a difference between the two phases in the patient with cerebellar stroke. The patient with cortical stroke described needing help with mobility and feeding as detailed on the Barthel assessment. Scoring on the composite VFQ was 72 versus 93 (cerebellar/cortical). The patient with cerebellar stroke had scores for both SARA and ICARS that indicated impairments in gait, tandem walking and standing balance with eyes closed.

**Table 1:** Results of the assessments

Assessments	Cerebellar	Cortical
FMA^a^	66/66	56/66
NHPT^b^ Rt (sec)	22	22
NHPT Lt (sec)	24	600
BNB^c^ Rt (#blocks/min)	59	50
BNB Lt (#blocks/min)	57	25
Barthel	100	85
Edinburgh handedness Before Stroke	Right	Right
Edinburgh handedness After Stroke	Right	Right
VFQ^d^ Composite Scores	73	93
Beery VMI^e^	27/30	26/30
ICARS^f^	8	–
SARA^g^	3	–

^a^ FMA: Fugl-Meyer Assessment, ^b^ NHPT: Nine Hole Peg Test, ^c^ BNB: Box and Blocks test, ^d^ VFQ: Visual Functioning Questionnaire, ^e^ Beery VMI: Beery-Buktenica Developmental Test of Visual-Motor Integration, ^f^ ICARS: International Cooperative Ataxia Rating Scale, ^g^ SARA: Scale for Assessment and Rating Ataxia

We investigated eye-hand coupling via: 1) eye and hand movement latencies, 2) eye and hand movement accuracy, 4) spatial and temporal variance, and 4) number of saccades (Table 2). Hand movement latencies were nearly identical in both patients (about 750 ms), but there were large differences in saccade latencies and their standard deviations, with the cortical stroke patient showing substantially longer latencies but more stability than the cerebellar stroke patient. The median number of saccades made during each reach was 4 (SD: 1.29) in the cerebellar stroke patient and 1 (SD: 1.3) in the cortical stroke patient. Eye spatial errors were larger in cerebellar than in cortical stroke. Hand spatial errors were larger in cortical than in cerebellar stroke.

**Table 2:** Results of the eye hand coordination experiments

Metrics		
	Cerebellar	Cortical
Spatial Error (hand; mm)	13.3 SD = 23.8	30.2 SD = 12.6
Spatial Error (eye; mm)	38.4 SD = 53.8	13.6 SD = 37.7
Number of Saccades	4 SD = 1.3	1 SD = 1.29
Hand Latency (ms)	752 SD = 179	742 SD = 165
Saccade Latency (ms)	261 SD = 776	451 SD = 172
Latency Difference ∆ = hand – eye (ms)	491 SD = 836	291 SD = 245

## Discussion

In this study, the onset of eye and hand movements were temporally decoupled with significant decoupling variance in the patient with cerebellar stroke, whereas the patient with cortical stroke displayed increased hand spatial errors and less decoupling variance. Increased decoupling variance in the patient with cerebellar stroke was primarily due to unstable saccade timing. This instability of saccadic latencies, which included both extremely short and very long latencies, resulted in a lower overall average saccadic latency in the cerebellar patient compared to the cortical patient. These findings highlight intriguing facets of eye-hand dyscoordination in these different lesion locations. Broadly speaking, our results corroborate the general notion that the cerebellum is instrumental to the process of temporal prediction for eye and hand movements, while the cortex is instrumental to the process of spatial prediction [5, 22, 28], both of which are critical aspects of functional movement control.

Even basic point-to-point visually-guided reaching is not a simple information transfer from perception to action but rather requires complex information processing, reflecting the correspondence between visually encoded spatial positions and arm coordinates. As such, visually guided arm movement starts with the transformation of the representation of a localized target from its initial coding in retinal coordinates to a body-centered frame of reference [29]. This body-centered frame of reference is more compatible with the intrinsic coordinates provided by the muscle, joint and skin receptors of the limb. The cerebral network that orchestrates this EHC-relevant activity is embedded within a widely distributed fronto-parietal network [30, 31].

The first step of this visually encoded spatial target transformation process, dependent on robust sensory input, is thought to be circumscribed within the parietal lobe and related to interactions between the superior and inferior parietal lobules (SPL / IPL) [30]. The benefit of multiple connections between SPL and IPL and dorsal and lateral frontal areas is the ability to process eye and hand information in parallel [31]. The outcome of parieto-frontal processing is eye and hand information that is matched to the coordinates needed in their respective motor output domains. The eye-hand network can be broken down neuroanatomically into 5 domains: posterior parietal, anterior parietal, cingulate, frontal and prefrontal cortex [30]. Near the parieto-occipital junction (POJ), the hand-eye integration domain (HEID) of the posterior parietal cortex (PPC), more specifically area Opt (lateral PPC) and the dorsomedial areas V6A and 7 m, contains critical nodes for binding retinal, eye and hand signals. While V6A and 7 m are the main sources of visual input to the arm-dominant SPL domain and the medial intraparietal area (MIP – a subdivision of area 5), area Opt projects to IPL, the eye-dominant domain, including the ventral intraparietal area (VIP), sensitive to motion, and the medial superior temporal area (MST) [29].

The cortical stroke in Case 2 suggests an impairment in eye-hand coordination that may have resulted from a disrupted connection between the frontal lobe and the superior parietal lobe neural network. Patients with cortical stroke have shown significant variability in the neuroanatomical regions that affect aspects of visually-guided reach performance post-injury. Lesions in both frontal and parietal lobes can create a constellation of limb impairments that include optic ataxia, directional hypokinesia, bradykinesia and hypometria [32], all of which may relate to the visual control of reaching. While variability has been noted in lesion location, it has been suggested that attention should be focused on independent assessment of the ocular motor and manual motor systems, i.e., the eye and hand, and their relationship [28, 32]. Although investigations in humans have been limited, and knowledge regarding interactions between parietal cortices and frontal motor areas is especially poor, new evidence has suggested that enhanced connectivity between the primary motor cortex and the anterior intraparietal sulcus correlates with enhanced recovery [33, 34].

As related to Case 1 and the role of the cerebellum in EHC, a previous review and study by Miall et al. [6] suggests that the cerebellum provides a forward model for the motor system, which is used to predict movements generated by a given control signal during movement planning. In this role, the forward model would create time-specific signals forecasting the motion of each motor effector and creating the necessary ‘blueprint’ required to plan the control signals needed for coordinated movement control. This study demonstrated improved manual tracking when eye and hand followed the same spatial trajectory and additional improvement when the eye led the hand by 75 to 100 ms. These findings suggest that the interaction between ocular and manual control systems are synergistic and optimal when dependent on synchronized action or temporal coupling; in addition, spatial congruence between trajectories or spatial coupling of ocular/visual feedback with manual control signals was also beneficial [6].

Imaging studies further support the concept that the cerebellum plays an important role in visually guided tracking; many suggest that this role is dependent on functions that utilize an internal clock and feed-forward computations to enable predictive control [35–37]. A number of regions in the cerebellum are involved in the temporal processing of information. In fact, the deep nuclei of the cerebellum are involved in ‘timing’ circuits with the basal ganglia (REF- Bares). There is also significant involvement in the right cerebellum during timed visual discrimination tasks and the left lateral cerebellum during timed movement generation tasks [35]. Studies have demonstrated increased activation between cerebellar vermis lobuli, hemispheric lobuli, posterior hemispheres and premotor and parietal areas when performing eye-hand reaching movements, as compared to isolated eye or hand movements [8, 38]. Previous reports have also shown impaired dexterity (measured by 9HPT) in patients with virtual cerebellar lesions using repetitive transcranial magnetic stimulation [39]. In non-human primates, cerebellar lesions result in more saccadic eye movements when following a target during a smooth pursuit task, as well as decreased correlations of eye and hand movement [40].

Our patient with cerebellar stroke displayed shorter, but substantially more variable, saccade latencies, corresponding in turn to larger-variance temporal onset differences between eye and hand movements. This finding appears broadly consistent with previous studies showing impaired temporal coupling of eye-hand movement in patients with cerebellar lesions [6]. The results of our study also show an increased number of saccades in the patient with cerebellar stroke, which is consistent with findings from Sailor et al., in which patients with cerebellar lesions, as compared to controls, make more saccades to reach the target than controls, but an equal number of visually triggered saccades [5]. This may suggest that inappropriate saccades could be suppressed during the preparation of goal-directed saccades in cerebellar lesions [6].

A detailed study of two patients reporting impaired eye-hand coordination after stroke, one cerebellar and one cortical, supports the perspective that different cortical and cerebellar contributions to normal eye-hand function may result in separable deficits following injury. In a case series, it is difficult to generalize the results to larger populations. Nevertheless, the findings observed here indicate that a larger study with appropriate statistical power could yield significant insight.

## Summary

In conclusion, we found that the conspicuous EHC deficits displayed by a patient with cortical stroke were primarily spatial, whereas those of a patient with cerebellar stroke were primarily temporal. Future studies should assess the spatial and temporal errors of EHC in acute and chronic cerebellar and cortical strokes, especially strokes identified to have subregions affected relevant to EHC, expanding our perspective on multi-effector coordination and cortico-cerebellar cooperation. Additionally, advanced MRI and non-invasive brain stimulation should be performed to detect and further characterize the neural networks involved in eye-hand coordination and dyscoordination.

## Data Availability

Not applicable.

## Abbreviations

EHC: Eye–Hand Coordination
NYU: New York University
FMA: Fugl-Meyer Assessment
NEI-VFQ-25: The 25-item National Eye Institute Visual Functioning Questionnaire
9HPT: Nine Hole Peg Test
BNB: Box and Blocks
ICARS: International Cooperative Ataxia Rating Scale
SARA: Scale for Assessment and Rating Ataxia
VOR: Vestibulo-Ocular Reflex
MRI: Magnetic Resonance Imaging
SD: Standard Deviation
SPL: Superior Parietal Lobule
IPL: Inferior Parietal Lobule
POJ: Parieto-Occipital Junction
HEID: Hand-Eye Integration Domain
PPC: Posterior Parietal Cortex
MIP: Medial IntraParietal
VIP: Ventral IntraParietal
MST: Medial Superior Temporal
VMI: Visual-Motor Integration

## References

[1] Rizzo J-R, Hosseini M, Wong EA (2017). The intersection between ocular and manual motor control: eye–hand coordination in acquired brain injury. Front Neurol.

[2] Carlton LG (1981). Visual information: the control of aiming movements. Q J Exp Psychol.

[3] Leigh RJ, Zee DS. The neurology of eye movements*.* Vol 90: Oxford University Press, USA; 2015.

[4] Thach WT, Goodkin HP, Keating JG (1992). The cerebellum and the adaptive coordination of movement. Annu Rev Neurosci.

[5] Sailer U, Eggert T, Straube A (2005). Impaired temporal prediction and eye-hand coordination in patients with cerebellar lesions. Behav Brain Res.

[6] Miall RC, Reckess GZ (2002). The cerebellum and the timing of coordinated eye and hand tracking. Brain Cogn.

[7] Coiner B, Pan H, Bennett ML (2019). Functional neuroanatomy of the human eye movement network: a review and atlas. Brain Struct Funct.

[8] Nitschke MF, Arp T, Stavrou G, Erdmann C, Heide W (2005). The cerebellum in the cerebro-cerebellar network for the control of eye and hand movements--an fMRI study. Prog Brain Res.

[9] Filippopulos F, Eggert T, Straube A (2013). Effects of cerebellar infarcts on cortical processing of saccades. J Neurol.

[10] Ranalli PJ, Sharpe JA (1986). Contrapulsion of saccades and ipsilateral ataxia: a unilateral disorder of the rostral cerebellum. Ann Neurol.

[11] Prsa M, Thier P. The cerebellum: eye movements. Neuroscience in the 21st Century New York*,* NY*:* Springer Science*.* 2013.

[12] Bodranghien F, Bastian A, Casali C (2016). Consensus paper: revisiting the symptoms and signs of cerebellar syndrome. Cerebellum..

[13] Tatler BW, Wade NJ, Kwan H, Findlay JM, Velichkovsky BM (2010). Yarbus, eye movements, and vision. i-Perception.

[14] Crawford JD, Medendorp WP, Marotta JJ (2004). Spatial transformations for eye-hand coordination. J Neurophysiol.

[15] Gaymard B (2012). Cortical and sub-cortical control of saccades and clinical application. Rev Neurol (Paris).

[16] Rizzo J-R, Fung JK, Hosseini M (2017). Eye control deficits coupled to hand control deficits: eye–hand incoordination in chronic cerebral injury. Front Neurol.

[17] Culham JC, Kanwisher NG (2001). Neuroimaging of cognitive functions in human parietal cortex. Curr Opin Neurobiol.

[18] Fugl-Meyer AR, Jaasko L, Leyman I, Olsson S, Steglind S (1975). The post-stroke hemiplegic patient. 1. A method for evaluation of physical performance. Scand J Rehabil Med.

[19] Sanford J, Moreland J, Swanson LR, Stratford PW, Gowland C (1993). Reliability of the Fugl-Meyer assessment for testing motor performance in patients following stroke. Phys Ther.

[20] Malloy P, Belanger H, Hall S, Aloia M, Salloway S (2003). Assessing visuoconstructional performance in AD, MCI and normal elderly using the beery visual-motor integration test. Clin Neuropsychol.

[21] Zagar R, Mead JD (1983). Analysis of a short test battery for children. J Clin Psychol.

[22] Serrien DJ, Wiesendanger M (2000). Temporal control of a bimanual task in patients with cerebellar dysfunction. Neuropsychologia..

[23] Feys P, Lamers I, Francis G (2017). The nine-hole peg test as a manual dexterity performance measure for multiple sclerosis. Mult Scler J.

[24] Mathiowetz V, Volland G, Kashman N, Weber K (1985). Adult norms for the box and block test of manual dexterity. Am J Occupational Therapy.

[25] Raphael BA, Galetta KM, Jacobs DA (2006). Validation and test characteristics of a 10-item neuro-ophthalmic supplement to the NEI-VFQ-25. Am J Ophthalmol.

[26] Cano SJ, Hobart JC, Hart PE, Korlipara LVP, Schapira AHV, Cooper JM (2005). International cooperative Ataxia rating scale (ICARS): appropriate for studies of Friedreich's ataxia?. Movement Disorders.

[27] Kim BR, Lim JH, Lee SA (2011). Usefulness of the scale for the assessment and rating of Ataxia (SARA) in ataxic stroke patients. Ann Rehabil Med.

[28] Bekkering H, Sailer U (2002). Commentary: coordination of eye and hand in time and space. Prog Brain Res.

[29] Caminiti R, Ferraina S, Mayer AB (1998). Visuomotor transformations: early cortical mechanisms of reaching. Curr Opin Neurobiol.

[30] Battaglia-Mayer A, Caminiti R (2018). Parieto-frontal networks for eye-hand coordination and movements. Handb Clin Neurol.

[31] Caminiti R, Innocenti GM, Battaglia-Mayer A (2015). Organization and evolution of parieto-frontal processing streams in macaque monkeys and humans. Neurosci Biobehav Rev.

[32] Battaglia-Mayer A, Archambault PS, Caminiti R (2006). The cortical network for eye-hand coordination and its relevance to understanding motor disorders of parietal patients. Neuropsychologia..

[33] Zimerman M, Weide S, Wessel M, Schulz R, Timmermann JE, Bönstrup M, Morishita T, Koch P, Gerloff C, Hummel FC. V37. Interactions between primary and secondary motor areas for recovered hand functions after stroke. Clin Neurophysiol. 2015;126(8):e84–5.

[34] Schulz R, Koch P, Zimerman M (2015). Parietofrontal motor pathways and their association with motor function after stroke. Brain.

[35] Aso K, Hanakawa T, Aso T, Fukuyama H (2010). Cerebro-cerebellar interactions underlying temporal information processing. J Cogn Neurosci.

[36] Miall RC, Reckess GZ, Imamizu H (2001). The cerebellum coordinates eye and hand tracking movements. Nat Neurosci.

[37] Miall RC, Jenkinson EW (2005). Functional imaging of changes in cerebellar activity related to learning during a novel eye–hand tracking task. Exp Brain Res.

[38] Miall RC, Imamizu H, Miyauchi S (2000). Activation of the cerebellum in co-ordinated eye and hand tracking movements: an fMRI study. Exp Brain Res.

[39] Liepert J, Hamzei F, Weiller C (2004). Lesion-induced and training-induced brain reorganization. Restor Neurol Neurosci.

[40] Vercher J-L, Gauthier GM (1988). Cerebellar involvement in the coordination control of the oculo-manual tracking system: effects of cerebellar dentate nucleus lesion. Exp Brain Res.

